# A Victory

**Published:** 1871-05

**Authors:** 


### PART IV.

### Editorials, Reviews, Items and News.

### A VICTORY.

Victory is associated in our minds with the idea of war. At the very mention of the word, there rises up before the imagination a great military hero, to whom tongues offer praise, and hearts spontaneously yield the homage which bravery and success will ever command. Amid the shouts that attend his pathway, we cannot hear the groans of the wounded and dying, nor the mourning from the homes made desolate. Through the cloud of incense that rises around him, we cannot discern cities wrapped in flames, and fields changed from smiling fetility into wastes by the tramp of the war horse. But we know that these scenes would form a pait and the greater part of the panorama of his life. We know that to kill and destroy has been his business, and that his success is nothing more than a display of signal ability to perform these. While we join in the shout as the wreath of fame is placed upon his brow, we shudder as we think of the human woe which it has lost. But there are other heroes than those of the battle field, and other victories than those won with the cannon and bayonet. It has indeed been elsewhere than in scenes of war that the highest types of heroism have been exhibited, and we should not name Marathon, Waterloo and Sedan as the greatest victories in the world's history. There are victories of Science, when some great principle in the laws of nature is developed by the brain of genius, and established as truth despite the opposition of ignorance and bigotry. Such a triumph was it when the now universally accepted theory of our Solar System received first the assent and then the admiring credence of the philosophic world. The great Florentine, who for asserting the fact of the earth's motion had been visited by the persecutions of the church, and by the ridicule of those who could not appreciate his discourses, lived not to witness this triumph. But every lover of truth must join in rendering honor to the name of one who labored and suffered in its behalf. Such a fame is more to be coveted and should be more lasting than the blood-stained wreathes with which the warrior invests his brow.

There are victories which deserve to be highly prized, which are more unobtrusive. The pioneer who enters the primeval forest and in a few years shows fields rich in crops and flocks, where but a little while since the wilderness was unbroken, merits no slight meed of praise for his success. The man who with poverty for his heritage, has accumulated a competence; who, by his own industry and energy has made for himself a home and a position in society; who has brought up a family of honest sons and virtuous daughters, has indeed achieved a success of which he may well be proud. The man who has persistently striven with the stormy and perverse elements within his own heart until he has brought them into subjugation, so that his daily life exemplifies the sublime virtues of the Christian character, has achieved a victory greater by far than if he had brought all the nations of the earth under his sway. There are triumphs which we can record with unmixed pleasure. They cost no suffering—they entail no misery. In the winning there are blessings brought, not upon the victor alone, but upon all with whom he is brought in contact.

The world is too much inclined to consider every success a victory, and too little disposed to *scrutinize* the means by which it has been attained. Nothing is worthy of the name of victory unless it be a noble end attained by worthy means. No power, no wealth, no fame can bring any sincere joy to the heart if they come as the results of arts that have degraded the moral nature. There wlll in the end be the bitterness and humiliation of failure. No laudation from others can conceal from the person himself the fact that in his efforts to succeed he has lowered the dignity of his moral nature and placed himself beyond the hope of recovery. Fame is at best but a poor compensation for the loss of self-respect. Wealth is no source of happiness when the soul has been pinched and dwarfed in its acquirements. Power gained by the sacrifice of princple cannot but degrade its possessor in his own estimation. Such victories are not advances upward. Too much is lost in gaining them. One must feel as did Pyrrhus after his first great battle with the Roman armies, that many such triumphs would bring ruin.

These successes of bad principles or of bad men do not indeed deserve to be rated as victorious at all. They contribute nothing for the true elevation, either of the individual or of society. On the contrary, they tend to the degredation of both; for by surrounding wickedness with a glamor, they make it more alluring and more destructive. By giving business position and prominence, they render it less detestable than it would otherwise seem. It is indeed a public misfortune when a bad man seems crowned with success in his efforts for power or fame. The influence of such an example will affect injuriously all but the bravest and purest hearts. Woe unto the land where the wicked reign; not solely because of the injustice and oppression that they will directly commit, but because when the bad triumph, many will despair of virtue and conclude that vice is far more profitable. Thus a great safeguard of society is broken down and the people, following the example of their rulers, give themselves up to immorality. It follows as an inevitable consequence that when the teachers are men of corrupt principles, the masses will be corrupt.

But the triumph of wrong cannot be lasting. It requires the constant aid of force or fraud to maintain its conquests. There is in truth an inherent energy which cannot be wholly subdued, and which is even ready to assert itself. When pressed down under the strong arm of tyranny, it is constantly exciting the suspicions of error, and threatening its empire with destruction. Dominion founded on wrong is always insecure. Though it may have power to burn, to torture, and to imprison its enemies, it will still feel as if the ground were giving way beneath its feet. When error rises to the ascendant, there is none of the proud confidence of victory. There is much of the feeling of the assassin, who, though secretly gloating in the vengeance he has wrought, is yet quaking with fear lest speedy justice may overtake him. There is a fear of proclaiming "we have a grand victory," lest men should be led to imagine what it is that has triumphed, and how that triumph has been attained. Let not the lover of truth, then, despond when he sees error in the ascendant. It will be but for a season. The Psalmist has most beautifully described the evanescent nature of the reign of error. He beheld the wicked flourishing like a green bay, filling a large space with his shadow, and to the passing observer might have promised long life and fruitfulness. But in a short time all was gone, and no trace left of its existence. Such are always the triumphs of the wicked. They are unsubstantial and uncertain. They may not always seem so. Often has the down-trodden and oppressed deemed the reign of error long. So long that the flickering ray of hope became well nigh extinguished. But the day of downfall came. The right long suppressed was vindicated, and wrong visited with its just retribution.

Victory in every struggle is desirable. Nor should any honest means for its attainment be neglected, retaining through every trial and every conflict our consciousness of rectitude. To fail is painful, but it cannot be disgraceful if we fail with honor untarnished, and principle uncompromised. Far better is it thus to fail, than to succeed by base and unworthy means. He who thus fails is a hero, and more than a hero—a martyr around whose name will linger for all ages a holy radiance, while not all the power that he may usurp, nor all the fame that he may acquire, will ever give a glorious immortality to the successful scheming villian.

